# Escaping the tragedy of the commons through targeted punishment

**DOI:** 10.1098/rsos.150223

**Published:** 2015-08-26

**Authors:** Samuel Johnson

**Affiliations:** Warwick Mathematics Institute, Centre for Complexity Science, University of Warwick, Coventry CV4 7AL, UK

**Keywords:** cooperation, social dilemma, climate change

## Abstract

Failures of cooperation cause many of society's gravest problems. It is well known that cooperation among many players faced with a social dilemma can be maintained thanks to the possibility of punishment, but achieving the initial state of widespread cooperation is often much more difficult. We show here that there exist strategies of ‘targeted punishment’ whereby a small number of punishers can shift a population of defectors into a state of global cooperation. We conclude by outlining how the international community could use a strategy of this kind to combat climate change.

## 1. Introduction

When Svante Arrhenius enunciated his greenhouse law in 1896, atmospheric concentration of CO_2_ stood probably at its highest in over half a million years—about 300 ppm [1,2]. It has now surpassed 400 ppm [3]. Our continued failure to avoid the well-known consequences of global warming is not rooted in some technical impossibility, but in a lack of international cooperation [4,5]. It is a classic example of the ‘tragedy of the commons’, as popularized by Garrett Hardin through the metaphor of herdsmen with access to common pasture land: each can always prosper individually by adding another head of cattle to his herd, but eventually this leads to overgrazing and ruin for all [6,7]. Other instances include overfishing, deforestation and many kinds of pollution. The solution advocated by Hardin was ‘mutual coercion, mutually agreed upon’, which is usually taken to mean coercion by a Hobbesian central authority [8,9]. Some argue that an alternative option is to ‘privatize the commons’ [10], although the coercion is still implicit in the assumption that property rights can be enforced [11]. Ironically perhaps, it is in local communities with access to some resource similar to Hardin's common pasture land where self-organization to cooperate has often been documented [12,13]. And indeed, such cases usually involve rules, mutually agreed upon, and enforced by the possibility of some form of punishment [14].

In game theory, the tragedy of the commons is seen as a Nash equilibrium, where no rational agent cooperates despite its being the strategy which would maximize collective payoff if adopted by all players^1^ [16]. Since cooperation is, nevertheless, pervasive in nature and society, much theoretical and empirical work has gone into understanding why this might be [17–23]. The focus has been on behaviour in the face of ‘social dilemmas’—situations where there is some communal benefit in choosing a cooperative strategy, but also a temptation to defect (not cooperate). In one-to-one games such as the *prisoner's dilemma*, a strategy of conditional cooperation can be individually advantageous if the game is iterated and players are able to remember each other [17,24]. A better model for commons management, however, is the *public goods game* [25,26]. Each player can choose how many tokens to put into a common pot which multiplies the total amount by some factor (greater than one and smaller than the number of players), and redistributes the result equally among all players. Many experiments with humans have shown that cooperation (i.e. adding to the magic pot) can be enhanced by allowing players to punish defectors, despite the punishers incurring a cost for doing so [20,27].

One aspect of social dilemmas which until recently has not been taken into account in theoretical studies is the heterogeneity of players [19]: even in laboratory experiments where the small number of subjects are all students, significantly different attitudes to cooperation are found [20]. In cases where the players are nation states, the differences are much larger. When it comes to tackling global warming, for instance, the heterogeneity in gross and *per capita* emissions, vulnerability to climate change, dependence on fossil fuels, historical responsibility, technical and financial ability to adapt, and many other relevant variables is widely regarded as confounding the problem [4]. However, recent work suggests that heterogeneity can in fact contribute to cooperation [28–31].

When public goods experiments are run in the laboratory, with the same group of subjects playing iteratively, cooperation tends to be high at first and gradually dwindle thereafter, possibly as cooperators become frustrated with the behaviour of defectors [20]. Allowing players to punish defectors from the start in such settings can discourage would-be defectors and maintain cooperation. In the real world, however, the problem is often not just one of maintaining cooperation, but of achieving it in an environment of almost ubiquitous defection. For instance, in a society with very little corruption, maintaining this happy state is relatively easy, since anyone attempting to break the rules would swiftly be identified and punished. In an environment of entrenched corruption, however, there are usually too many defectors and too few resources to change the state of affairs [32].

It is often assumed that punishment—and indeed positive incentives—must be seen as fair, and there is some evidence that unfair or inconsistent punishment fails to maintain cooperation in laboratory experiments [33]. But in situations of widespread defection, the punishing capacity of would-be punishers (usually a subset of cooperators), if applied equally to all defectors, can be too dilute to have any effect. Here we use a simple model to show that in such situations there may exist strategies of ‘targeted punishment’ which punishers can adopt in order to escape the tragedy of the commons and bring about universal cooperation. A degree of heterogeneity among the players will contribute to the strategy's effectivity—and may, perhaps, serve to assuage any feelings of unfairness.

## 2. Results

### 2.1 Maintaining versus achieving cooperation

Let us consider a set of *N* players faced with a social dilemma of some kind. At any given moment, each player can choose either of two strategies, or *states*: to cooperate or to defect.^2^ Depending on the details of the situation, a player will perceive a net payoff associated with each state. Let us call the difference of these perceived payoffs *H*_*i*_ for player *i*. Thus, if *i* has all the relevant information, and is entirely selfish and rational, she will cooperate if *H*_*i*_>0 and defect if *H*_*i*_<0. We shall consider, however, that the degree to which these assumptions hold can be captured by a ‘rationality’ parameter *β*, in such a way that *i* has, at each time step *t*, a probability *P*_*i*_ of cooperating and a probability 1−*P*_*i*_ of defecting, where

2.1 $$P_{i}=\frac{1}{2}[\tanh(\beta H_{i})+1].$$

This sigmoidal form coincides with the transition probabilities for the spins in an Ising model, or for the neurons in a Hopfield neural network [34], and is often used, as here, to model players of games [35,36]. Behaviour is completely random if *β*=0, and becomes deterministic (perfectly rational) when β→∞. The need to take this feature into account is suggested by work in evolutionary game theory and behavioural economics which has highlighted the importance of somewhat stochastic or bounded rationality [37–39].

What form shall we choose for *H*_*i*_? We are interested in situations where most of the players are individually predisposed to defect. However, these predispositions can be heterogeneously distributed. Let us assume, with no loss of generality, that the sequence *i*=1,2,…,*N* positions the players in order of their predisposition, from most to least intrinsically cooperative. For simplicity, let us consider that the predisposition *h*_*i*_ of player *i* is given by the linear expression *h*_*i*_=−(*i*−2)/(*N*−2). Thus, for any *N*, the first player is the only one with a slight tendency to cooperate, the second one has no inherent tendency, and each successive player has a greater tendency to defect than the previous one, down to the last with *h*_*N*_=−1. In addition to this individual effect, each player can be influenced by the others. For instance, let us assume that a certain number of players *n*_*p*_ have each a capacity *π* to punish defecting players they consider at fault, of which there are *n*_f_. The total punishment befalling a defector among the *n*_f_ is then *p*_*i*_=*πn*_p_/*n*_f_. The balance of payoffs for a given player considered at fault is now

2.2 $$H_{i}=p_{i}+h_{i}=\pi \frac{n_{p}}{n_{f}}-\frac{i-2}{N-2}.$$

(Note that *n*_p_ and *n*_f_ can change with time, although for clarity we refrain from making this explicit.) This is also the balance *H*_*i*_ for players who are cooperating but who would become at fault if they were to defect (with the small adjustment that, since such a player would presumably not punish herself, she has $p_{i}={\tilde{n}}_{p}/{\tilde{n}}_{f}$, where ${\tilde{n}}_{p}$ and ${\tilde{n}}_{f}$ are the values of *n*_p_ and *n*_f_ that there would be if this player defected). Meanwhile, for players not considered at fault irrespectively of their states, *p*_*i*_=0. We are assuming that the players know each other's states, as is usually the case, for instance, of governments choosing climate policy.

This simple model captures the features of social dilemmas required to illustrate how targeted punishment can work, without sacrificing generality by going into the details of a given game. However, the parameter *π* could be adjusted to describe, for instance, the public goods game with specific punishment costs.

Which players can punish, and whom should they punish? In many real situations, it is only possible for cooperators to punish defectors. In this case, *n*_p_ is equal to the number of cooperators, *n*_*c*_, at any given time. For now we shall focus on this case, although the possibility of defectors also punishing other defectors is discussed below.^3^ As to who should be punished, this is in fact the only ‘rule’ that the community has freedom to determine—or, more precisely, that those in a position to punish can determine. The simplest (and arguably fairest) rule would be for all defectors to be punished. In this case, *n*_f_ is equal to the number of defectors, *n*_f_=*N*−*n*_c_.

**Figure 1.**
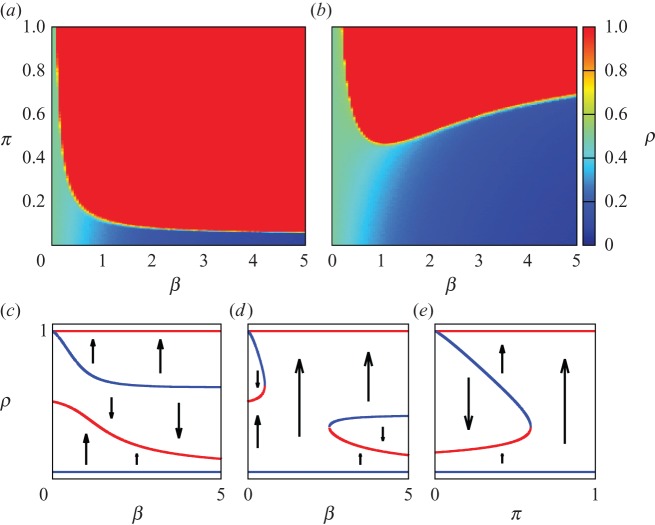
(*a*) Stationary proportion of cooperators, *ρ*, for a range of rationality, *β*, and punishment, *π*, from Monte Carlo simulations of the model when all cooperators punish all defectors, and initially all *N*=200 players cooperate. (Results are the averages over 100 realizations.) (*b*) As before, but now all players initially defect. (*c*) Fixed points of the dynamics against *β*, when *π*=0.4; stable fixed points are depicted in red, unstable ones in blue. (*d*) As in (*c*), but with *π*=0.6. (*e*) Fixed points of the dynamics against *π*, when *β*=2.5. (The fixed-point analysis is described in Methods.)

We run computer simulations of the situation described above for *N*=200 players (roughly the number of countries in the world) and compute the average proportion of cooperators, *ρ*=*n*_c_/*N*, once a stationary state has been reached. Figure 1 shows this proportion on a colour scale for a range of the two parameters, *β* (rationality) and *π* (punishment). In figure 1*a*, we see that for almost all parameter combinations global cooperation is obtained. However, there is an important detail: for these simulations, we have set the initial state of every player to ‘cooperate’. The lesson we can learn, therefore, is that in these conditions global cooperation can be maintained once it has been achieved. But what about if the initial states are all set to ‘defect’? In figure 1*b*, we show the results for this case. There is now a much smaller region of global cooperation, requiring significantly higher levels of punishment *π* than are necessary simply to maintain cooperation. Interestingly, while a certain degree of rationality *β* is needed to achieve cooperation, thereafter the minimum punishment enabling cooperation increases with rationality, implying that some degree of randomness in the selection of states is globally beneficial. This positive influence of stochasticity has also been observed in other settings, such as one-to-one games on graphs [41].

What is happening here? To gain a better understanding of the phenomenon, we consider the *fixed points* of the dynamics. These are values ρ* such that, when *ρ*=ρ*, the subsequent value to which the system naturally evolves is, again, ρ*. A fixed point can be either stable or unstable: if a small deviation from ρ* would tend to return the system to ρ*, it is stable; whereas it is unstable if random fluctuations around this point are amplified and the system driven to some other value of *ρ*. In Methods, we analyse the fixed points and their stability, and show the results for three different combinations of parameters in figure 1*c*−*e*. Figure 1*c* corresponds to a level of punishment *π*=0.4. The lines show the fixed points as functions of *β*, with stable fixed points plotted in red and unstable ones in blue. Arrows show the direction in which the system will tend to evolve depending on the value of *ρ* (away from unstable fixed points and towards stable ones). First of all, we observe that global defection (*ρ*=0) is always unstable, while global cooperation (*ρ*=1) is stable for any *β*>0. There are two further fixed points, one stable and one unstable. If the system begins with sufficient cooperators that *ρ* is above the unstable one, it will evolve towards global cooperation. However, if the initial *ρ* is below this, evolution will be towards the other stable fixed point. This explains the difference between the top two panels, where global cooperation is observed when all players begin cooperating, but not when they start off defecting. Figure 1*d* shows a situation of higher punishment, *π*=0.6. There are now still regions of *β* for which the stable fixed point at low *ρ* acts as a trap when all players begin defecting; but an interval has appeared in which there is an uninterrupted path from *ρ*=0 to *ρ*=1. This corresponds to the region in figure 1*b* where cooperation can be observed at this *π* for intermediate values of *β*. Finally, in figure 1*e* we set *β*=2.5 and plot the fixed points against *π*. Again we see that, for *π* below a certain value, there is a stable fixed point at low *ρ* which acts as a trap, while high enough *π* will ensure global cooperation irrespectively of initial conditions.

### 2.2 Paths to cooperation

**Figure 2.**
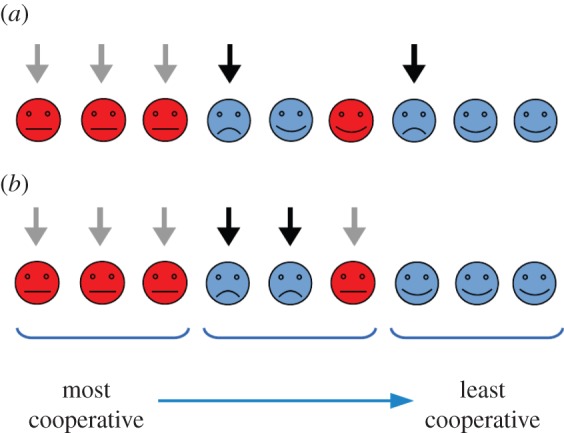
Diagrams illustrating the two strategies of targeted punishment described in the main text: (*a*) the ‘single file strategy’ and (*b*) the ‘groups strategy’ with groups of size *ν*=3 and a threshold *θ*=2/3. Players are arranged from most to least inherently cooperative; those currently cooperating are shown in red and those defecting in blue. A black arrow indicates a defector who is considered at fault (and therefore liable to be punished) according to the strategy, while a grey arrow signals a cooperator who would be at fault if she were defecting.

**Figure 3.**
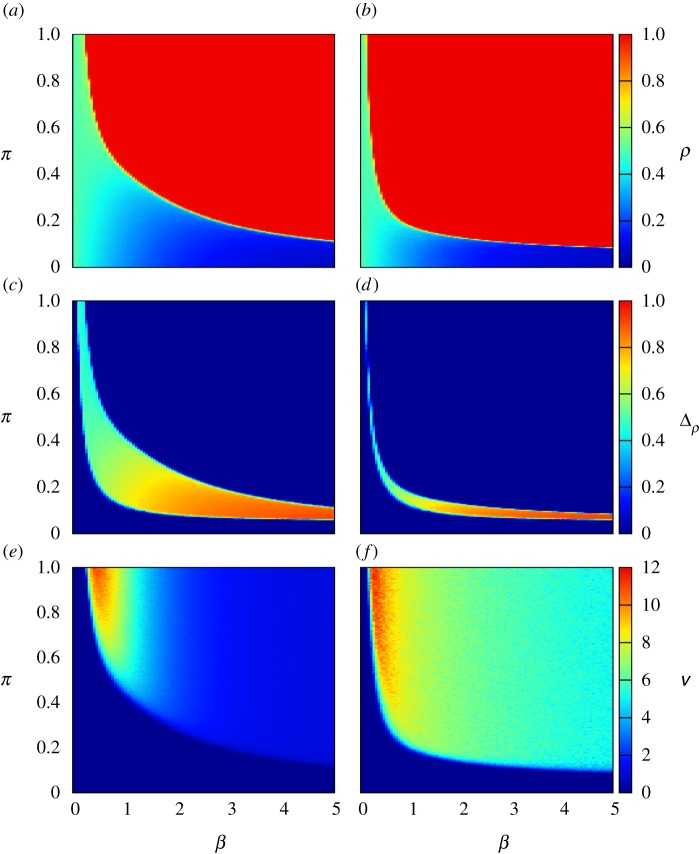
(*a*) As figure 1*b* (all players initially defect), but now the ‘single file strategy’ is applied. (*b*) As in figure 1*b*, but under the ‘groups strategy’ with *ν*=10 and *θ*=80%. (See the main text and figure 2 for descriptions of these strategies.) (*c*) Difference between figure 1*a* (all players initially cooperate) and figure 3*a*. (*d*) Difference between figures 1*a* and 3*b*. (*e*) Speed *v*=*N*/*τ*, where *τ* is the number of time steps required to achieve global cooperation, for the situation in figure 3*a*. (*f*) Speed *v* for the case of figure 3*b*.

As noted above, players with the ability to punish others have the freedom to decide whom to punish. It may seem fairest to punish all defectors equally, but when these are numerous this approach dissipates the total punishing capacity. Consider, instead, the following rule. A defecting player *i* is only deemed at fault at time *t* if the one immediately before her in the ordering, player *i*−1, cooperates at time *t*. This rule, which we shall refer to as the ‘single file strategy’, is illustrated in figure 2*a*. According to this view, the number of players considered at fault, *n*_f_, will be smaller than the total number of defectors when these are in the majority, while the scenario becomes identical to the previous one when almost all the players cooperate. In figure 3*a*, we show the results for simulations in which punishers adopt this strategy. As in figure 1*b*, all players initially defect. The region of global cooperation is now significantly larger than in figure 1*b*: harmony can be achieved at much lower values of punishment *π*, particularly if rationality *β* is high. Because at any one time only a very small number of defectors are deemed at fault, even a low level of punishment is sufficient to make them cooperate. As each new player switches state, it passes on the burden of responsibility to another one down the line, resulting in a cascade of defectors becoming cooperators. A secondary effect is that, as the ranks of cooperators grow, the total punishment they are able to inflict increases, although as we show in the electronic supplementary material this is not essential for the mechanism to work.

The single file strategy allows for global cooperation to ensue from widespread defection in situations where this would not have been possible with equal allocation of punishment. Thus, selecting only certain players for culpability can provide an escape route from the tragedy of the commons. However, this is not necessarily the best rule to ensure such an outcome; in fact, there are regions at low *π* and *β* where, according to figure 1, global cooperation is sustainable, yet not achievable via this route. So consider now the following arrangement, which we can call the ‘groups strategy’. Players are allocated to groups of size *ν*, such that players *i*=1,…,*ν* belong to the first group, *i*=*ν*+1,…,2*ν* to the second, and so forth. A defector belonging to group *m* is deemed to be at fault at time *t* if and only if at least a proportion *θ* of the players in group *m*−1 cooperate at time *t*. This strategy is illustrated in figure 2*b*. In figure 3*b*, we show simulation results for this scenario, with 20 groups of *ν*=10 players and a threshold of *θ*=80%, where, as before, all players begin defecting. (To set the process off, players in the first group are always considered at fault if they defect.) An even greater region of parameter space now corresponds to global cooperation, leaving only very low levels of *π* and *β* out of reach. For a given number of players, *N*, and set of inherent tendencies, *h*_*i*_, there will be optimal rules, or ‘targeted punishment strategies’, which come closest to ensuring global cooperation for any values of rationality and punishment. (Note that the single file strategy is an instance of the more general groups strategy when *ν*=1 and *θ*>0.)

Figure 3*c*,*d* shows the difference, Δ_*ρ*_, between the maximum density of cooperators achievable (i.e. when all players initially cooperate), and the results of figure 3*a*,*b*, respectively. About 12% of the parameter space shown corresponds to situations where cooperation is possible but not achievable via the single file strategy. For the groups strategy, however, little over 3% of the potential parameter space remains out of reach. These results are obtained by considering the initial conditions ‘all defect’, since this is the worst case scenario: any other initial conditions, such as a given fraction of players cooperating, will have at least equal probability of ending in global cooperation. Another aspect to take into account when comparing punishment strategies is the speed with which cooperation can be achieved. Figure 3*e*,*f* shows the quantity *v*=*N*/*τ* for each rule, where *τ* is the number of time steps required to achieve cooperation. In most of the parameter range, cooperation is achieved sooner with *ν*=10 than with *ν*=1.

As remarked above, in many real situations it is only the cooperators who are seen as having the ability or legitimacy to punish defectors. However, defectors too could, in principle, punish other defectors, even if this may be regarded as somewhat unfair. For instance, in many societies criminals pay value added tax on their purchases, thereby contributing indirectly to the penal system; and possessing nuclear weapons does not keep countries from using sanctions to discourage proliferation elsewhere. In the electronic supplementary material, we perform the same analysis for the case in which *n*_p_=*N*; that is, all players contribute to the punishment of defectors. The dynamics is qualitatively similar to the situation in which only cooperators can punish, the main difference being that global cooperation can, unsurprisingly, ensue from lower levels of punishment per player. If, on the other hand, only a fraction *a* of cooperators were to punish, the situation would be as in figures 1 and 3 after rescaling π→aπ.

The situations thus far examined involve a predisposition to cooperate, *h*_*i*_, with a specific functional form; and the punishing strategies assume that their precise ordering is known. In the electronic supplementary material, we relax these constraints by adding a Gaussian noise to *h*_*i*_, and randomly switching the ordering of 25% of players with randomly chosen counterparts. We find that both punishing strategies described above are quite robust to these changes: the single file strategy is the most robust at high levels of both punishment and rationality, while the groups strategy is superior at low values of these parameters. When only cooperators can punish, the mechanism is most effective if players are positioned in order of their predispositions, since those most predisposed to cooperate require fewest cooperators/punishers to change their states. When total punishment is independent of the number of cooperators, however, the ordering has little effect. We also carry out simulations for *N*=1000 players and show that the results are not contingent on a small system size.

Finally, we have seen that an ordering, or grouping, based on the heterogeneity of natural predispositions {*h*_*i*_} leads to effective targeted punishment—but is heterogeneity necessary? Monte Carlo simulations with varying degrees of heterogeneity (while maintaining constant mean predisposition) show that targeted punishment provides a path to cooperation even when all players have equal predisposition, but a degree of heterogeneity is a contributing factor. This is in keeping with other recent work on the role of diversity in cooperation [28–31].

Targeted punishment is an example of indirect reciprocity. It is also reminiscent of a spatial structure, or some form of assortment—another of the established mechanisms conducive to cooperation in evolutionary games [19]. It differs from the standard instantiations of the latter, however, in two respects: the structure is strategically designed by the players in the absence of exogenous constraints; and the structure does not determine who plays with whom, but rather who is punished.

## 3. Discussion

Punishment has been shown to maintain cooperation in many social dilemma settings [20,27], and it is generally assumed that such punishment should be fair [33]. However, in situations of entrenched defection, the society's punishing capacity can become too dilute to have any effect if applied equally to all defectors. The message of this paper is that in such situations there may exist strategies of ‘targeted punishment’ which allow a few initial punishers to shift a large number of defectors into a state of global cooperation.

The paths to cooperation described above would seem to rely heavily on the possibility of punishment. Since punishing a defector presumably has some cost, such an act in itself constitutes a kind of cooperation. This is not necessarily a problem, given that humans and governments alike are wont to engage in ‘costly punishment’ in a variety of settings [20,27,42]. But, in any case, punishment is only one potential mechanism which might give rise to a term *p*_*i*_ with the characteristics we have here assumed. For instance, a determining factor in human behaviour often seems to be the anticipation of how one's choices might affect those of others [23], or one's perception of risk [5]. We know that our recycling, voting or travelling by bicycle will have little impact on the world *per se*, but we may rationally engage in these activities in the hope that others will follow suit. If the rules of the game have been set up in such a way that our actions determine whether the next player in line will be expected to honour her conditional commitments, what seemed like a grain of sand in the desert becomes a grain of sand in an avalanche. Imagining all eyes turned towards the single player whose turn it is to cooperate—or to the single small group of such players—it is easy to see how one might be more inclined to cooperate than in a world of distributed responsibility.

One could argue that establishing the initial ordering would be an obstacle of similar magnitude to achieving cooperation directly. This may be the case when the players are alike in all respects. But an acknowledgement of heterogeneity might break this symmetry in a way acceptable to all, especially if there exist objective measures to establish, say, the effort each player would have to make to cooperate. Furthermore, a player fairly well inclined to cooperate but deterred by the mass of less well-predisposed companions might happily adopt an early position in the hope that the mechanism may bear fruit; while staunch defectors can leave the burden of responsibility to others by being placed further down the line, with the knowledge that they would only be called upon to participate if global cooperation were nigh. In any case, if the tool for convincing players to cooperate is some form of punishment, only the punishers need agree on whom to punish at any given time.

Tavoni and colleagues have developed a model in which players consume a common resource subject to ‘equity-driven ostracism: a denial of support by the cooperating community which has tangible consequences on the wealth of the norm violators’ [43], p. 159. This mechanism is similar to the punishment considered here in that its effects are greater the smaller the number of (punishable) defectors. It would be interesting to consider the effects of targeted punishment in this setting.

It is worth reflecting that much social organization as we know it is in fact achieved through an implicit arrangement of targeted punishment. Even the most despotic tyrants cannot personally punish all dissenters. But if they can exert power over a small group of underlings, who in turn manage their subordinates, and so on down a hierarchical pyramid, top-down control can occur. Similarly, most of us are subject to the judging gazes of only our immediate friends and neighbours, yet this can be enough to ensure conformity to various social conventions. The moral to draw might be ‘punishment begins at home’.

### 3.1 Targeted punishment in practice

Could a strategy of targeted punishment be implemented by the international community to escape from global tragedies of the commons, such as anthropogenic climate change? Ideally, countries might sign up voluntarily to small groups, each of which would in turn be allocated a position in an ordering. Alternatively, the ordering and groupings could follow automatically from some objective measure, such as income *per capita*. Small nations already making significant yet unsung progress, or particularly vulnerable ones, could use their early positioning to draw attention to their situations in the hope of having a wider effect. Others may initially welcome the temporary lifting of responsibility by signing up to a group further down the line, but find themselves obliged to cooperate once the spotlight came their way. Finally, even the biggest polluters would run out of excuses once a majority of other groups were cooperating. Some combination of sanctions and incentives could be arranged, although the mere fact of the whole world's eyes being focused on a small number of defectors at any one time might prove a sufficient inducement in many cases. This kind of scheme would be consistent with recent suggestions to divide nations into groups in order to augment the perception of risk and reduce uncertainty of outcome [5]. The ingredient we propose here to add is simply the interaction between groups. We have already tried signing up to commitments, enshrining these in law, privatizing the commons through carbon trading schemes—yet yearly global CO_2_ emissions are now about 30% higher than when the Kyoto Protocol was adopted [4,44]. Perhaps it is time for a new approach.

## 4. Methods

According to equations (2.1) and (2.2), the probability that player *i* will cooperate at time step *t*+1 is

$$P_{i}(t+1)=\frac{1}{2}\tanh\left[\beta \left(\pi \frac{n_{p}(t)}{n_{f}(t)}-\frac{i-2}{N-2}\right)\right]+\frac{1}{2},$$

where *n*_p_(*t*) and *n*_f_(*t*) are the numbers of punishing and punishable players, respectively, at time *t*. If *ρ*_*t*_=*n*_c_(*t*)/*N* is the proportion of cooperating players at time *t*, let us define the expected proportion of cooperating players at time *t*+1: $G(\rho_{t})={\bar{\rho }}_{t+1}$ (this is an expected value in the sense that the average of *ρ*_*t*+1_ over many independent realizations of the system will converge to ${\bar{\rho }}_{t+1}$). We can then write

$$G(\rho_{t})=\langle P_{i}(t+1)\rangle ,$$

where 〈⋅〉 stands for an average over all players. For the case where *n*_p_=*n*_c_ and *n*_p_=*N*−*n*_c_ (all defectors are punished by, and only by, all cooperators), this becomes

4.1 $$G(\rho_{t})=\frac{1}{2}\left\langle \tanh\left[\beta \left(\pi \frac{\rho_{t}}{1-\rho_{t}}-\frac{i-2}{N-2}\right)\right]\right\rangle +\frac{1}{2}.$$

Any value ρ* such that *G*(ρ*)=ρ* will be a fixed point of the dynamics. Fluctuations around ρ* will tend to dampen out if

4.2 $${\left.\frac{dG(\rho_{t})}{d\rho_{t}}\right|}_{\rho_{t}=\rho^{*}}\in (-1,1),$$

whereas if the absolute value of the derivative is larger than one, the fixed point will be unstable, since even an infinitesimal fluctuation will drive the system to a different state. Figure 1*c*−*e* is obtained by solving equations (4.1) and (4.2) numerically. More generally, for any *ρ*_*t*_, it is possible to determine whether the system can be expected to evolve towards more or fewer cooperators by the sign of *G*(*ρ*_*t*_)−*ρ*_*t*_.

## Supplementary Material

Supplementary Material for “Escaping the Tragedy of the Commons through Targeted Punishment”: In this appendix we test the robustness of our results by relaxing some of the assumptions made in the main text.

## Footnotes

1 Note, however, that this depends on one's definition of ‘rational’: for Kant, not cooperating here would violate the categorical imperative, and therefore defy reason [15].

2 We use here the word ‘state’ for this variable to distinguish it from the strategies of targeted punishment.

3 There is no reason, in principle, why defectors should not be able to retaliate by punishing the punishers, a behaviour known as ‘antisocial punishment’ [40]. However, since the focus here is on situations where all players recognize the collective benefits of cooperation, as in the case of global warming, we shall leave this possibility unexplored.
