# Supplementary material for: Escaping the tragedy of the commons through targeted punishment
Source: R Soc Open Sci. 2015 Aug 26;2(8):150223. doi: 10.1098/rsos.150223 (PMC4555856; doi:10.1098/rsos.150223)
Supplement: Supplementary Material for “Escaping the Tragedy of the Commons through Targeted Punishment”: In this appendix we test the robustness of our results by relaxing some of the assumptions made in the main text. [file rsos150223supp1.pdf]

SUPPLEMENTARY MATERIAL
for
Escaping the Tragedy of the Commons through Targeted Punishment

Samuel Johnson
Warwick Mathematics Institute, and Centre for Complexity Science,
University of Warwick, Coventry CV4 7AL, United Kingdom.
E-mail: S.Johnson.2@warwick.ac.uk

In the main text we describe two strategies of targeted punishment which punishers can adopt when most of the players are defecting:

- Single file: A defector is considered at fault if and only if the player immediately before her in the ordering is currently cooperating.
- Groups: A defector is considered at fault if and only if at least a fraction θ of the players making up the group immediately before hers in the ordering are currently cooperating.

The strategies are most effective if players are arranged in descending order of their net perceived payoff h_i – that is, in increasing order of their temptation to defect. We show that by focusing their punishment on defectors considered at fault according to the rule adopted, punishers can shift a population of defectors towards global cooperation in many situations where attempting to punish all defectors would have no appreciable effect. But the generality of these results is limited by two assumptions: that the number of punishers is proportional to the number of cooperators; and that the perceived payoffs of players follow a linear form, which is known to punishers. In this appendix we relax both assumptions and find that the effectivity of targeted punishment does not depend strongly on such considerations (Sections 1 and 2). We also look into the effects of the number of players (Section 3) and of heterogeneity (Section 4). Our results are found not to depend on a small system size, and heterogeneity is not necessary for targeted punishment to work, although it does promote cooperation.

1 Constant punishment

In the main text, we consider only scenarios in which the number of punishers is equal to the number of cooperators. If it were, in fact, proportional to the number of cooperators, this would simply involve a rescaling of the punishment parameter, π. But what if all players were punishers, irrespectively of their individual state of cooperation? Figure S1 shows the situations corresponding to

Figure 1 of the main text, with the difference that now the number of punishers is $n_p = N$ at all times. As in the case where only cooperators punish, there is a large region of parameter space where cooperation is sustainable, but not achievable when punishment is diluted among all defectors.

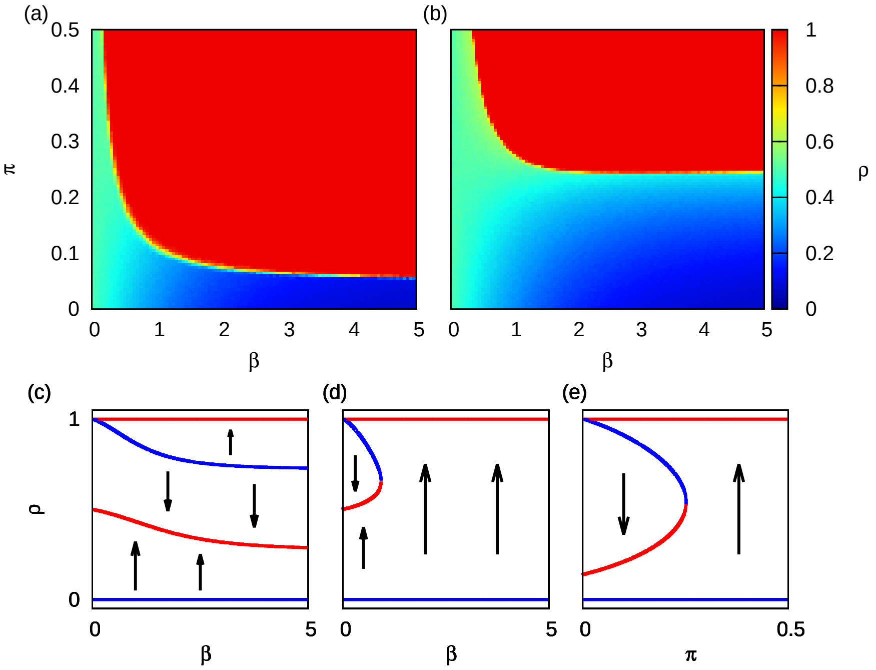

Figure S 1. As Figure 1 of the main text, but with the number of punishers being equal to the number of players: $n_p = N$. (a) All players initially cooperate. (b) All players initially defect. Bottom panels: Stability diagrams when $\pi = 0.2$ (c), $\pi = 0.3$ (d), and $\beta = 2.5$ (e).

The targeted punishment strategies described above are able to bring about global cooperation in large regions of the parameter space, as can be seen in Figure S2 – this figure corresponds to Figure 3 of the main text, with the difference that here $n_p = N$, instead of $n_p = n_c$. Note that, if we wished to consider a situation where a fraction a of players where punishers, it would suffice to rescale the punishment parameter as $\pi \to a\pi$ in Figures S1 and S2.

2 Robustness to noise

In the main text we consider situations where player i's payoff, in the absence of punishment, is $h_i = -(i-2)/(N-2)$, and players are arranged in descending order of h. In real situations, punishers may not know the payoff perceived by player i. We therefore corrupt this setting with two sources of noise to gauge the strategies' robustness to this imperfect knowledge. We now consider that i's payoff is $h_i = -(i-2+\eta_i)/(N-2)$, where the variables $\{\eta_i\}$ are drawn from

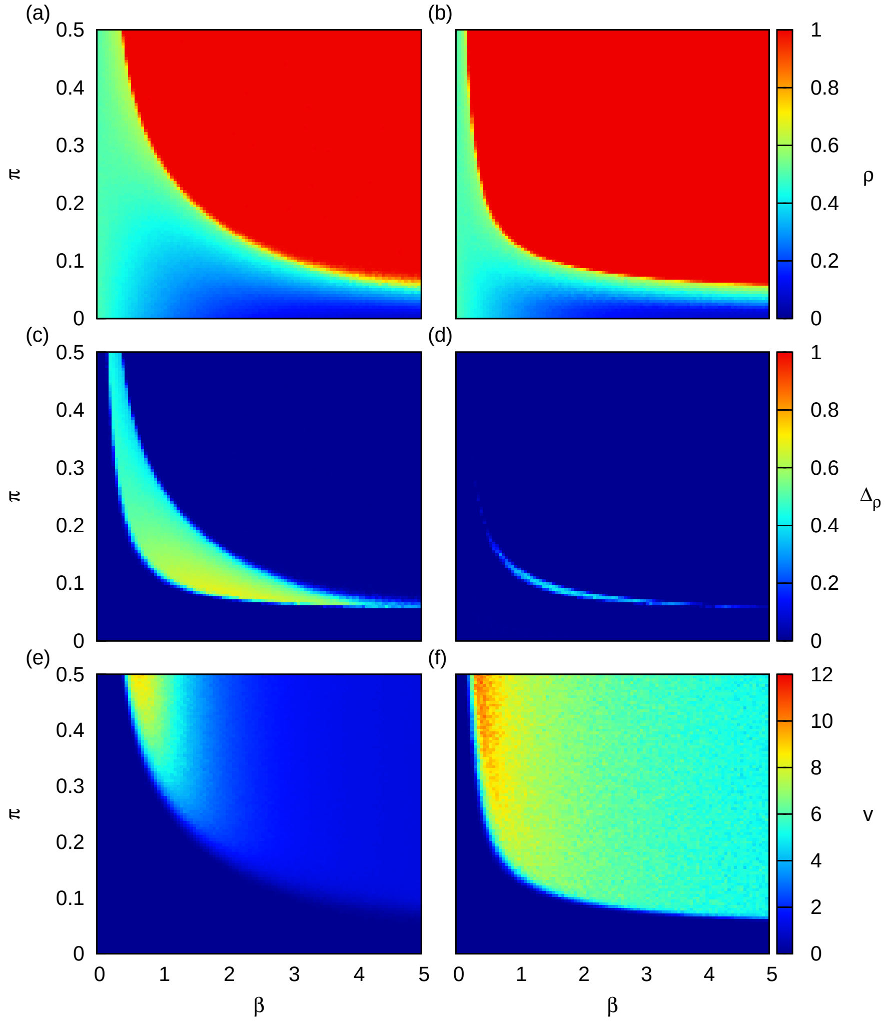

Figure S 2. As Figure 3 of the main text, but with the number of punishers being equal to the number of players: $n_p = N$. (a) As Figure S1b (all players initially defect), but now the 'single file strategy' is applied. (b) As in Figure S1b, but under the 'groups strategy' with $\nu = 10$ and $\theta = 80\%$. (c) Difference between Figure S1a (all players initially cooperate) and Figure S2a. (d) Difference between Figure S1a and Figure S2b. (e) Speed $v = N/\tau$, where τ is the number of time steps required to achieve global cooperation, for the situation in Figure S2a. (f) Speed v for the case of Figure S2b.

a Gaussian with mean zero and variance σ^2. We also reshuffle the ordering, by choosing a fraction f of the N players randomly, and switching their positions in the ordering with randomly chosen players. Figure S3 shows a setting like that of Figure 3 of the main text, after we have corrupted the payoffs with a (quenched) noise set by $\sigma^2 = 1$, and reshuffled a proportion $f = 25\%$ of players.

Although the targeted punishment strategies lose some of their effectivity, there are still large regions of parameter space where their adoption leads to most players cooperating.

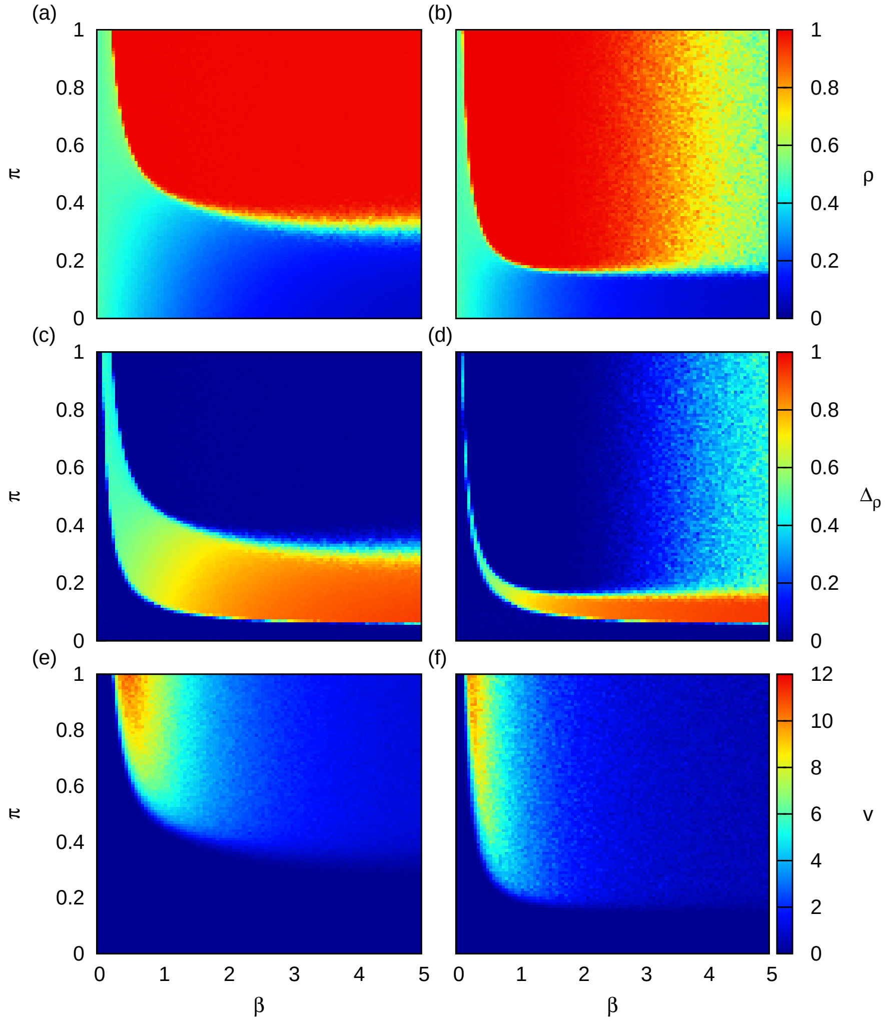

Figure S 3. As Figure 3 of the main text, after the payoffs have been corrupted with a noise drawn from a Gaussian of mean zero and variance $\sigma^2 = 1$; and a random proportion $f = 25\%$ of players have had their positions in the ordering switched with other random players. Punishment strategies are 'single file' in the panels on the left [(a),(c) and (e)], and 'groups' in those on the right [(b),(d) and (f)]. (a) and (b) Stationary proportion of cooperators, ρ. (c) and (d) Difference between maximum ρ maintainable [as displayed in Figure 1(a) of the main text], and the results of Figures S3a and S3b, respectively. (e) and (f) Speed $v = N/\tau$, where τ is the number of time steps required to achieve global cooperation, for the situations in Figures S3a and S3b, respectively.

In Figure S4, we carry out the same corruption of payoffs and of the ordering as in Figure S3, but here we consider the situation where all players are punishers: $n_p = N$. Again, the strategies can be seen to be fairly robust under these conditions.

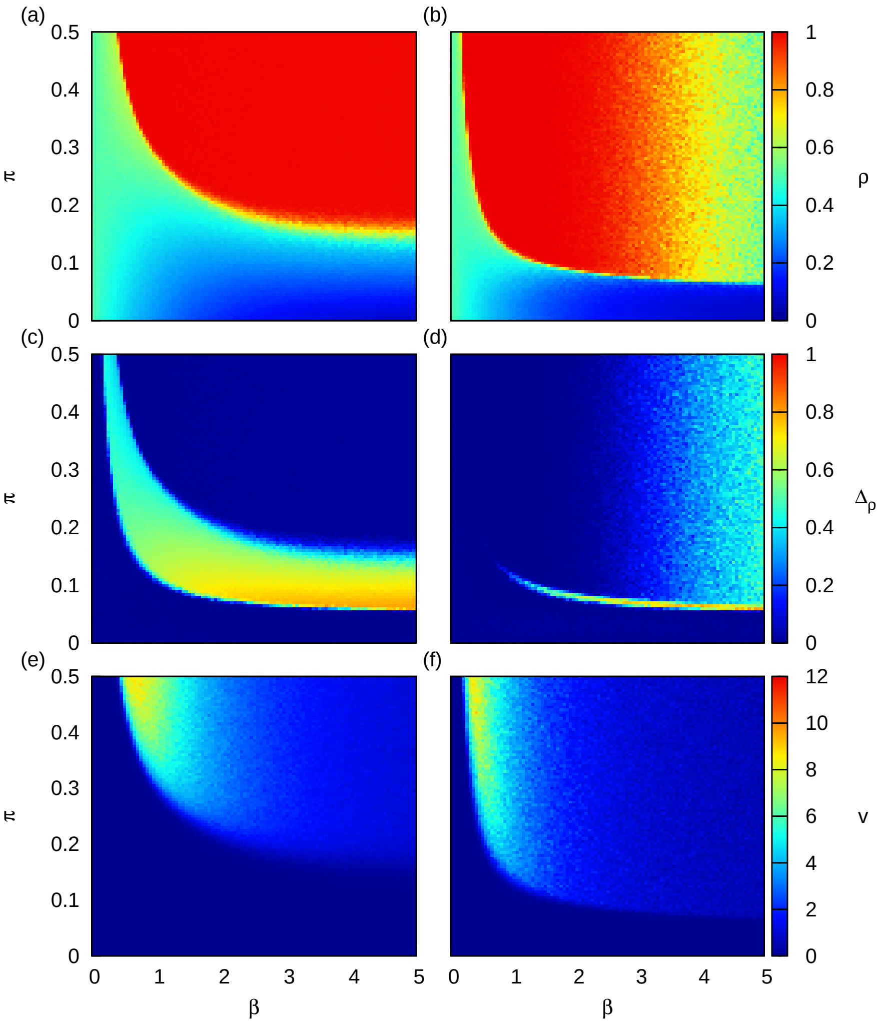

Figure S 4. As Figure S2 (all players punish), after the payoffs have been corrupted with a noise drawn from a Gaussian of mean zero and variance $\sigma^2 = 1$; and a random proportion $f = 25\%$ of players have had their positions in the ordering switched with other random players. Punishment strategies are 'single file' in the panels on the left [(a),(c) and (e)], and 'groups' in those on the right [(b),(d) and (f)]. (a) and (b) Stationary proportion of cooperators, ρ. (c) and (d) Difference between maximum ρ maintainable [as displayed in Figure S1a], and the results of Figures S4a and S4b, respectively. (e) and (f) Speed $v = N/\tau$, where τ is the number of time steps required to achieve global cooperation, for the situations in Figures S4a and S4b, respectively.

3 Number of players

The results presented thus far in the figures are for a relatively small set of players, $N = 200$, a value chosen to coincide roughly with the number of countries in the world, and to make the heat-maps of previous figures computationally inexpensive. None of the reported results ensue from finite-size effects, however, and strategies of targeted punishment can work for any size N. In Fig. S5 we show the stationary proportion of cooperators against punishment level for $N = 1000$ players initially set to defect, for each of the punishment strategies described. There is always a discontinuous (first-order) transition between a regime in which global cooperation is attained, and one in which most players defect indefinitely. The targeted punishment strategies serve to limit the proportion of parameter space yielding coexistence of phases – in other words, they reduce the surface of the hysteresis loop.

The fact that our results are independent of system size suggests that strategies of targeted punishment might also be effective in situations where the players are a large number of individuals. For instance, if every person willing to recycle waste, vote or travel by bicycle chooses just a few individuals to chastise whenever they fail to cooperate too, socially beneficial behaviour can spread throughout society. Needless to say, under certain conditions, socially noxious behaviour might also percolate by the same mechanism.

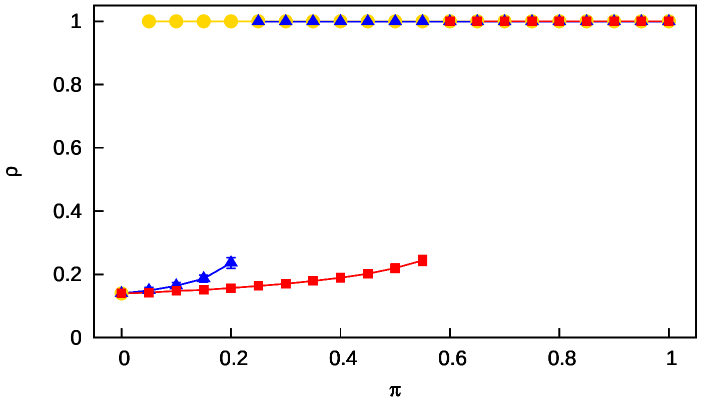

Figure S 5. Stationary proportion of cooperators, ρ, against punishment, π, for $N = 1000$ players with initial conditions 'all defect', and rationality $\beta = 2.5$. Red squares: equal punishment (no strategy). Blue triangles: 'single file strategy'. Yellow circles: 'groups strategy', with 100 groups of 10 players, and a threshold $\theta = 80\%$. In all cases, the number of punishing players coincides with the number of cooperators. Averages are over 100 Monte Carlo realizations, with standard deviations shown as error bars (in most cases, smaller than the symbols).

4 Heterogeneity

We have so far been considering situations in which the individual predispositions of players are heterogeneously distributed. In the absence of noise, these have been given by $h_i = -(i-2)/(N-2)$. We expect the effectivity of targeted punishment strategies to be increased by heterogeneity, since it allows cooperation to build up gradually, beginning with the players most inclined to cooperate. In order to look into the importance of this aspect, we consider a different set of predispositions, $\{\tilde{h}_i\}$, defined as $\tilde{h}_i = \alpha h_i + (1-\alpha)\langle h\rangle$, where $\langle h\rangle = N^{-1}\sum_i h_i = [2-(N+1)/2]/(N-1)$ is the mean predisposition, and α is a parameter determining the degree of heterogeneity: for $\alpha = 0$ the situation is completely homogeneous, while for $\alpha = 1$ we recover the heterogeneity considered in the main text. Note that the mean predisposition is independent of α.

In Fig. S6 we show the effect of heterogeneity on each of the punishment strategies. When punishment is applied equally to all defectors, heterogeneity has no effect on the stationary proportion of cooperators, ρ. Under strategies of targeted punishment, heterogeneity does indeed increase the parameter range in which global cooperation can be achieved. However, the strategies are still significantly more effective than equal punishment even for completely homogeneous predispositions ($\alpha = 0$). We can therefore conclude that heterogeneity is not necessary for targeted punishment to work. It is noteworthy, however, that in the 'groups strategy' case not all Monte Carlo runs achieve global cooperation when $\alpha = 0$. The proportion which do seems to depend on the maximum time the simulation is run for (in Fig. S6, 10^4 MCS), suggesting that global defection acts as a metastable state from which the system eventually escapes thanks to a degree of irrationality (finite β).

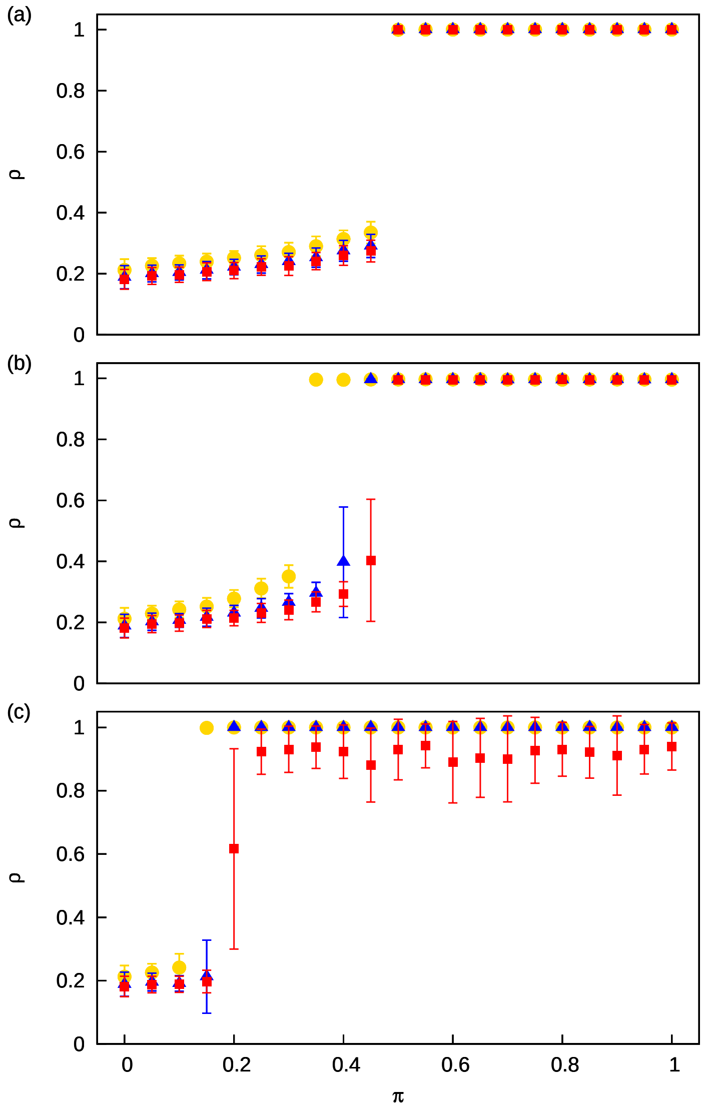

Figure S 6. (a) Stationary proportion of cooperators, ρ, against punishment, π, for $N = 200$ players with initial conditions 'all defect', rationality $\beta = 1.5$, and varying degrees of heterogeneity according to symbols. Red squares: $\alpha = 0$ (no heterogeneity). Blue triangles: $\alpha = 0.5$. Yellow circles: $\alpha = 1$ (heterogeneity as in main text). (b) As in (a), but under the 'single file strategy'. (c) As in (a), but under the 'groups strategy', with 20 groups of 10 players, and a threshold $\theta = 80\%$. In all cases, the number of punishing players coincides with the number of cooperators. Averages are over 50 Monte Carlo realizations, with standard deviations shown as error bars.
